# Effect of Lactational Low-Protein Diet on Skeletal Muscle during Adulthood and Ageing in Male and Female Mouse Offspring

**DOI:** 10.3390/nu16172926

**Published:** 2024-09-01

**Authors:** Moussira Alameddine, Atilla Emre Altinpinar, Ufuk Ersoy, Ioannis Kanakis, Ioanna Myrtziou, Susan E. Ozanne, Katarzyna Goljanek-Whysall, Aphrodite Vasilaki

**Affiliations:** 1 Department of Musculoskeletal & Ageing Science, Institute of Life Course & Medical Sciences (ILCaMS), University of Liverpool, Liverpool L7 8TX, UK; moussira.alameddine@manchester.ac.uk (M.A.); a.e.altinpinar@liverpool.ac.uk (A.E.A.); ersoyuf@liverpool.ac.uk (U.E.); ikanakis@liverpool.ac.uk (I.K.); kwhysall@liverpool.ac.uk (K.G.-W.); 2 Division of Developmental Biology and Medicine, Faculty of Biology, Medicine and Health, University of Manchester, Manchester M13 9PT, UK; 3 Chester Medical School, Faculty of Health, Medicine and Society, University of Chester, Chester CH1 4BJ, UK; i.myrtzioukanaki@chester.ac.uk; 4 MRC Metabolic Diseases Unit, Welcome Trust-MRC Institute of Metabolic Science, Addenbrooke’s Treatment Centre, Addenbrooke’s Hospital, University of Cambridge Metabolic Research Laboratories, Cambridge CB2 0QQ, UK; seo10@cam.ac.uk; 5 Department of Physiology, School of Medicine and REMEDI, CMNHS, University of Galway, H91 TK33 Galway, Ireland

**Keywords:** low-protein diet (LPD), skeletal muscle, ageing, lactation, sarcopenia

## Abstract

Sarcopenia is characterised by the loss of skeletal muscle mass and function, which leads to a high risk of increased morbidity and mortality. Maternal malnutrition has been linked to impaired development of skeletal muscle of the offspring; however, there are limited studies that report the long-term effect of a maternal low-protein diet during lactation on the ageing of skeletal muscles. This study aimed to examine how a maternal low-protein diet (LPD) during lactation affects skeletal muscle ageing in the offspring. Pups born from control mothers were lactated by mothers fed with an LPD. Post-weaning, mice were either maintained on an LPD or switched to a control, normal-protein diet (NPD). In males, an LPD mainly affected the size of the myofibres without a major effect on fibre number and led to reduced grip strength in ageing mice (24 months). Female mice from mothers on an LPD had a lower body and muscle weight at weaning but caught up with control mice at 3 months. During ageing, the muscle weight, myofibre number and survival rate of female pups were significantly affected. These findings highlight the effect of an LPD during lactation on skeletal muscle ageing, the lifespan of offspring and the importance of sexual dimorphism in response to dietary challenges.

## 1. Introduction

Skeletal muscles develop in early embryological stages when the number of fibres is determined [1]. Moreover, muscle mass and muscle fibre number depend on the proliferation and maturation of muscle precursor cells [2]. It has been demonstrated that low muscle mass is linked to all-cause mortality risk, and skeletal muscle mass can serve as a mortality predictor in the elderly [3,4,5]. The late initiation of a differentiation of precursor muscle cells leads to a bigger muscle mass at birth [2]. In humans, the loss of muscle fibres generally starts after the age of 30, and the number of myofibres significantly decreases around the age of 70, leading to the loss of muscle strength and function, thus leading to frailty [6,7]. Age-related sarcopenia typically includes the loss of muscle strength and function in addition to reduced muscle mass [8,9,10], but the underlying mechanisms have not been fully identified. However, studies suggest that factors such as a sedentary lifestyle and poor maternal diet play a role in inducing muscle loss [7,11]. It has also been shown that poor maternal diet during early life stages negatively affects overall growth and has a long-term effect on the offspring [12,13].

Proteins are the building units of skeletal muscles; therefore, dietary amino acid uptake through proteins is essential in maintaining the growth and repair of muscle mass and function [14,15]. Previous studies have shown that protein restriction during lactation inhibited the development of mammary glands, ultimately causing a decrease in the quantity and quality of breast milk produced [16,17,18]. This inadequacy of milk supply leaves the new-borns malnourished, causing delays in their growth and a decrease in their overall body weight, which can have short-term and long-term effects [16,17,18]. This decrease in body weight and delayed growth of offspring is associated with sarcopenia and several metabolic diseases, such as type 2 diabetes mellitus [12,19,20,21,22,23]. Furthermore, studies have reported that short-term protein restriction during lactation (i.e., normalised protein intake post-weaning) in humans and rodents led to muscle wasting and reduction in muscle strength, muscle weight and muscle fibre diameter [22,24,25,26]. In addition, previous studies showed a correlation between longevity and induced slow growth rate of offspring caused by a lactational low-protein diet (LPD) [27,28,29]. Our group has shown that a postnatal low-protein diet led to a decrease in gastrocnemius and soleus muscle weight and a decrease in the number of myofibres of solei of male pups during ageing [30]. However, the effects of a low-protein diet during lactation on survival rate and skeletal muscle (tibialis anterior) in male and female pups during ageing have not been previously established.

A lactational LPD can have a different effect on skeletal muscle for male and female offspring [31]. Systems regulating energy metabolism differ between males and females, leading to different responses to the maternal diet [31]. Other factors such as hormones, adipokines, placenta and epigenetics also play a role in sexual dimorphism in maternal diet studies [32]. Different hormonal regulation can be one of the main factors explaining the differences in the lifespan and response of males and females to maternal diet [31].

Mice show analogous loss of muscle fibres and whole motor units over their lifespan to humans [33]. The aim of this observational study was, therefore, to investigate the life-long effects of a lactational LPD on muscle mass and function of male and female progenies. We hypothesised that a protein-deficient diet during lactation would promote muscle loss and would lead to an early onset of sarcopenia.

## 2. Materials and Methods

### 2.1. Animals and Experimental Design

B6.Cg-Tg(Thy1-YFP)16Jrs/J mice (The Jackson Laboratory; *Thy-1 YFP-16*, Stock# 003709) were housed in the Biomedical Service Unit (BSU) at the University of Liverpool and subjected to a 12 h light–12 h dark cycle. All experiments described here were ethically approved by the University of Liverpool Animal Welfare and Ethical Review Body (AWERB) and performed in accordance with the UK Home Office guidelines under the UK Animals (Scientific Procedures) Act 1986. All experimental work was performed under a designated project licence granted by the UK Home Office (PPL code: P4A0493B2; approval date: 26 March 2019).

Female mice were fed either a normal (N)-protein diet (20% Crude Protein W/W ISO’ (P), Code 829206, Special Diet Services, Essex, UK) or a low (L)-protein diet (8% Crude Protein W/W ISO’ (P), Code 829202; Special Diet Services, Essex, UK) and were mated with male mice fed a normal-protein diet. The females were fed their respective diets from 2 weeks before mating and throughout mating, birth and lactation. The normal- and low-protein diets were isocaloric.

**Figure 1:**
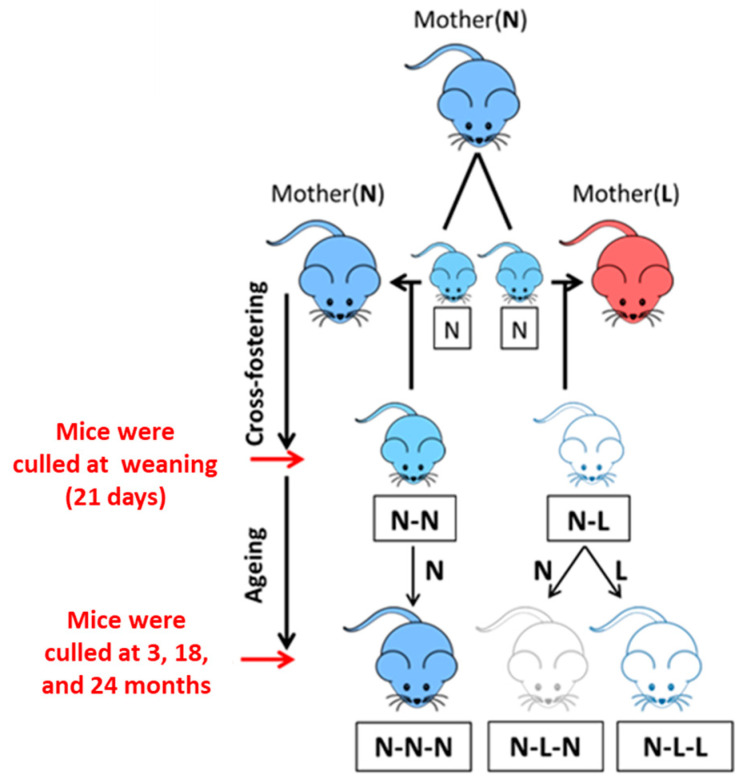
The experimental design of (N) 20% diet and (L) 8% diet. Group N: Control mice produced from a mouse dam fed a normal-protein diet. Group N-N: Mice produced from a mouse dam maintained on a normal-protein diet and fed postnatally by a mouse dam maintained on a normal-protein diet. Culled and analysed at weaning (21 days). Group N-L: Mice produced from a mouse dam maintained on a normal-protein diet but fed postnatally by a mouse dam maintained on a low-protein diet. Culled and analysed at weaning (21 days). Group N-N-N: Control mice produced from a mouse dam fed on normal-protein chow, fed postnatally by a mouse dam maintained on a normal-protein diet until weaning and maintained on a normal-protein diet. Culled and analysed at 3 months, 18 months and 24 months. Group N-L-N: Control mice produced from a mouse dam fed with a normal-protein diet, fed postnatally by a mouse dam maintained on a low-protein diet until weaning and maintained on a normal-protein diet. Culled and analysed at 3 months, 18 months and 24 months. Group N-L-L: Control mice produced from a mouse dam fed on a normal-protein diet, fed postnatally by a mouse dam maintained on a low-protein diet until weaning and maintained on a low-protein diet. Mice were culled and analysed at 21 days, 3 months, 18 months and 24 months.

New-born pups born from a mother fed an NPD were cross-fostered and lactated by a dam fed either an NPD or an LPD, leading to two different groups of mice: NN and NL. The number of suckling pups was kept similar (*n* = 5–6) for both NPD and LPD lactating mouse dams. After 21 days, weanling mice were fed either an NPD or an LPD throughout their lifespan, leading to three different groups: NNN, NLN and NLL. NNN mice represent the offspring control group. NLN mice were fed an LPD only during lactation, and NLL mice were fed an LPD during their lifespan. Healthy mice were culled through a rising concentration of carbon dioxide (CO_2_) at 21 days (21 d), 3 months (3 M), 18 months (18 M) and 24 months (24 M) of age. Body weight was measured immediately after culling. NN and NNN groups served as controls for 21 d and 3 M/18 M/24 M, respectively (Figure 1).

To allow the calculation of appropriate mouse numbers for each of the studies, statistical power calculations were performed based on preliminary and published data. Thus, a total of 11 mice per group (per age) was originally allocated to complete the experiments. Note: The number of mice during ageing was different in some groups. This is because a significant number of mice developed illnesses, particularly during ageing, and had to be humanely killed. Therefore, the total number of mice (per group) used for this research was as follows: 9 male and 8 female mice for the NN group; 10 male and 8 female mice for the NL group; 25 male and 16 female mice for the NNN group; 14 male and 25 female mice for the NLN group; 18 male and 19 female mice for the NLL group. Due to the nature and complexity of the experimental design, the exact number of animals used for each analysis per age group is stated separately in each figure’s legend.

The number of mice used was kept to a minimum by performing multiple assessments on the same mouse. Mice were allocated at random for control and experimental groups. Mice were allocated to different groups during breeding and maintenance by our Biomedical Services Unit (BSU) staff at the University of Liverpool who were also responsible for monitoring welfare. Animals on low-protein diets were highlighted to enable extra vigilance for early signs of ill health. Appearance, behaviour and food and water intake were monitored daily. A scoring system was used to help with decision making and monitoring once the first signs of ill health were seen. Animals displaying the following signs of ill health were humanely culled: immobility, reduced food/water intake, laboured breathing, gait abnormalities that inhibit the animal’s ability to move around the cage and reach food/water and weight loss of more than 20% compared with near-age-matched controls.

### 2.2. Grip Strength

To analyse the effect of a maternal LPD on the physical performance of progenies during ageing, grip strength was measured. The grip strength of 18-month-old mice was assessed monthly using the four-limb hanging test. Mice were placed on a grid to acclimatise, and then the grid was inverted while the mice gripped using 4 limbs. The hanging time was recorded from the moment the grid was inverted until the mice lost their grip and fell onto a 5-to-7 cm soft bedding. The mice were left to rest and then the test was repeated as described in TREAT-NMD SOP DMD_M.2.1.004 [34]. The data were analysed as maximum hanging time and holding impulse (hanging time × body weight), taking into consideration the body weight of the mice.

### 2.3. Histological Analysis

The purpose of this experiment was to study the histological effect of a lactational LPD on the *Tibialis anterior* (TA) muscles of male and female progenies. *TA* muscles were dissected, weighed, processed and embedded in cryomatrix (Fisher Scientific, Loughborough, UK) and then immersed in iso-pentane (Fisher Scientific, Loughborough, UK), cooled in liquid nitrogen (LN2) for 30 s and stored at −80 °C.

Prior to sectioning, samples were left in the cryostat (Leica 1900, Leica Microsystems, Wetzlar, Germany), which was set at −20 °C for 30 min for acclimatisation. Sections of 10 µm thickness were collected on superfrost plus adhesion slides (Ct# J1800AMNZ, Fisher Scientific, Loughborough, UK). The sections were stored at −20 °C for staining.

Before staining, muscle sections were left at room temperature for 30 min to air-dry. The sections were washed with ice-cold phosphate-buffered saline (PBS) for 2 min to wash away the cryomatrix and then fixed with ice-cold methanol (MetOH; Cat# 34860, Sigma Aldrich, Dorset, UK) for 5 min at room temperature. The fixed sections were washed thrice with PBS-Tween 20 (0.04% *v*/*v*; Sigma Aldrich, Dorset, UK) for 5 min each. Muscle sections were incubated in 1:1000 wheat germ agglutinin (WGA—fluorescein 5 μg/mL; Cat# FL-1021-5, Vector Laboratories Ltd., Peterborough, UK) for 10 min, followed by three washes of PBS-T for 5 min each. The sections were washed with H_2_O and left to dry for a few hours. Finally, the sections were mounted with hard-set DAPI (4′, 6-diamidino-2-phenylindole—hard set; Cat# H-1500, Vector Laboratories Ltd., Peterborough, UK). The slides were left overnight in the dark at 4 °C. Before scanning, the slides were left at room temperature to dry, and then they were cleaned with 70% ethanol. The sections were scanned with a Z1 digital slide scanner (Carl Zeiss Microscopy, New York, NY, USA). Images were acquired using a 20× objective. Slides were always kept in the dark. Finally, images were analysed by using a fully automated system, MyoVision (University of Kentucky, Lexington, KY, USA), to measure the cross-sectional area (CSA) of the myofibres and count the number of fibres per section [35].

### 2.4. Statistical Analysis

**Figure 2:**
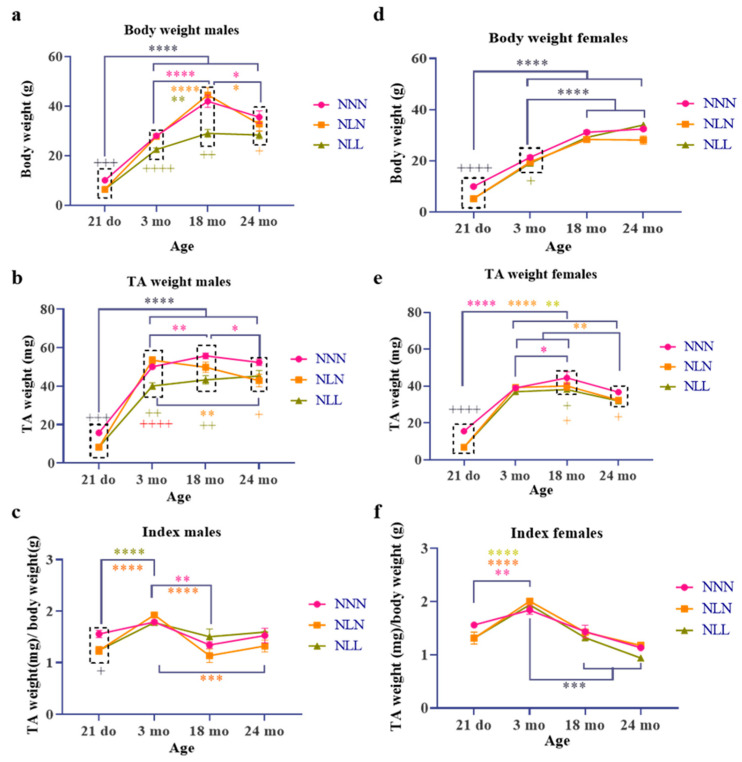
The effect of an LPD during lactation on body and muscle weight, and the index of male (**a**–**c**) and female (**d**–**f**) progenies. The effect of an LPD during lactation on the body weight of male mice throughout the lifespan of NNN, NLN and NLL (**a**). The effect of an LPD during lactation on the TA weight of male mice throughout the lifespan of NNN, NLN and NLL (**b**). The effect of an LPD during lactation on the index of male mice throughout the lifespan of NNN, NLN and NLL (**c**). The effect of an LPD during lactation on the body weight of female mice throughout the lifespan of NNN, NLN and NLL (**d**). The effect of an LPD during lactation on the TA weight of female mice throughout the lifespan of NNN, NLN and NLL (**e**). The effect of an LPD during lactation on the index of female mice throughout the lifespan of NNN, NLN and NLL (**f**). Data are presented as mean ± SEM at 21 days NN (*n =* 7 for males, *n =* 8 for females) and NL (*n =* 10 for males and *n =* 8 for females), at 3 months NNN (*n =* 6 for males and *n =* 8 for females), NLN (*n =* 7 for males and *n =* 8 for females) and NLL (*n =* 13 for males and *n =* 11 for females), at 18 months NNN (*n =* 6 for males and *n =* 5 for females), NLN (*n =* 3 for males and *n =* 9 for females) and NLL (*n =* 3 for males and *n =* 7 for females) and at 24 months NNN (*n =* 7 for males and *n =* 3 for females), NLN (*n =* 4 for males and *n =* 8 for females) and NLL (*n =* 2 for males and *n =* 1 for females), using one-way ANOVA followed by Tukey’s post hoc test. *** *p* ≤ 0.001 and **** *p* ≤ 0.0001 represent all groups; * *p* ≤ 0.05, ** *p* ≤ 0.01 and **** *p* ≤ 0.0001 represent the NNN group; ** *p* ≤ 0.01, *** *p* ≤ 0.001 and **** *p* ≤ 0.0001 represent the NLN group; ** *p* ≤ 0.01 and **** *p* ≤ 0.0001 represent the NLL group; + *p* ≤ 0.05, +++ *p* ≤ 0.001, ++++ *p* ≤ 0.0001 are used to compare NN and NL at 21 days of age; + *p* ≤ 0.05, ++ *p* ≤ 0.01, ++++ *p* ≤ 0.0001 are used to compare NNN and NLL at 3, 18 and 24 months of age; + *p* ≤ 0.05 is used to compare NNN and NLN at 3, 18 and 24 months of age; ++++ *p* ≤ 0.0001 is used to compare NLL and NLN at 3 months of age; dotted rectangles refer to the significant difference between the groups at a certain age; do refers to days old and mo refers to month old. No statistical analysis was performed on 24-month-old NLL females because only one mouse survived to this age.

**Figure 3:**
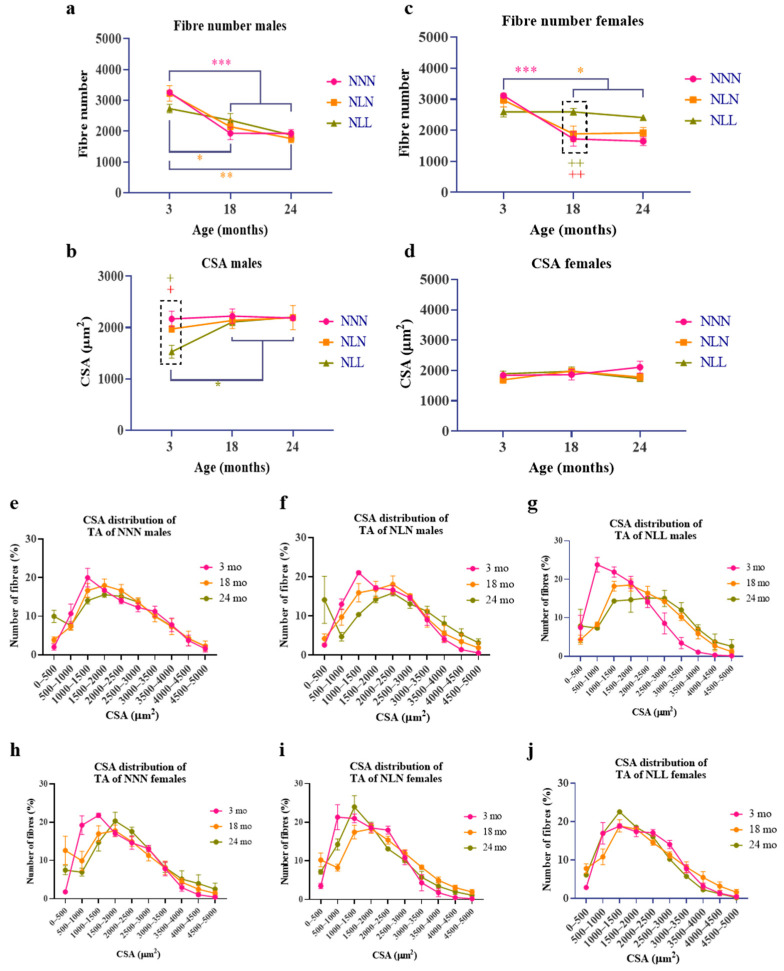
The effect of an LPD during lactation on the myofibres of the TA muscle of male (**a**,**b**,**e**,**f**) and female (**c**,**d**,**h**–**j**) progenies. The effect of an LPD during lactation on the total fibre number of the TA muscle of males (**a**) and females (**c**) throughout the lifespan of NNN, NLN and NLL. The effect of an LPD during lactation on the cross-sectional area (CSA) of the myofibres of the TA of males (**b**) and females (**d**) of NNN, NLN and NLL. The distribution of the myofibres of NNN, NLN and NLL of males (**e**–**g**) and females (**h**–**j**). Data are presented as mean ± SEM at 3 months NNN (*n =* 4 for males and females), NLN and NLL (*n =* 5 for males and females), at 18 months NNN (*n =* 4 for males and females), NLN (*n =* 4 for males and *n =* 5 for females) and NLL (*n =* 3 for males and *n =* 5 for females) and at 24 months NNN (*n =* 4 for males and *n =* 3 for females), NLN (*n =* 3 for males and *n =* 3 for females) and NLL (*n =* 2 for males and *n =* 1 for females), using one-way ANOVA followed by Tukey’s post hoc test (**a**–**d**) and the chi-square test and two-way ANOVA followed by Tukey’s post hoc test (**e**–**j**). *** *p* ≤ 0.001 represents the NNN group, * *p* ≤ 0.05 and ** *p* ≤ 0.01 represent the NLN group, and * *p* ≤ 0.05 represents the NLL group; + *p* ≤ 0.05 and ++ *p* ≤ 0.01 are used to compare NNN and NLL at 3 and 18 months of age, + *p* ≤ 0.05 and ++ *p* ≤ 0.01 are used to compare NLL and NLN at 3 and 18 months of age; dotted rectangles refer to the significant difference between the groups at a certain age; mo refers to months old. No statistical analysis was performed on 24-month-old NLL females because only one mouse survived to this age.

**Figure 4:**
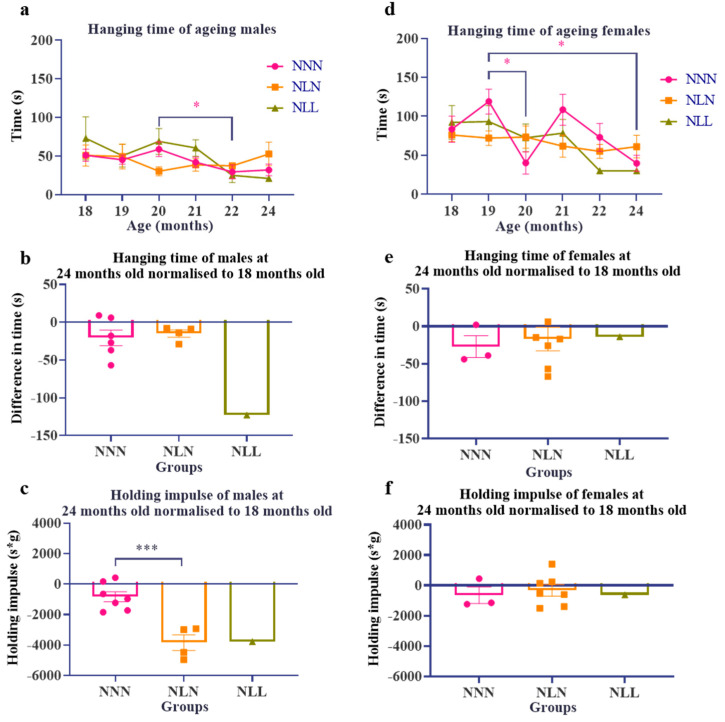
The grip strength of ageing male (**a**–**c**) and female (**d**–**f**) mice. Hanging time was measured as maximum hanging time and holding impulse (hanging time × body weight). The hanging time of ageing male (**a**) and female mice (**d**) (>18 months of age) of NNN, NLN, and NLL groups was measured monthly until the mice were 24 months of age. The hanging time of 24-month-old male mice was normalised to 18 months (**b**) and female mice (**e**). The holding impulse of 24-month-old male mice was normalised to 18 months (**c**) and female mice (**f**). Data are presented as mean ± SEM, for NNN male (*n =* 10 at 18 months and *n =* 6 at 24 months), NLN male (*n =* 6 at 18 months and *n =* 4 at 24 months), NLL male (*n =* 4 at 18 months and *n =* 1 at 24 months), NNN female (*n =* 5 at 18 months and *n =* 3 at 24 months), NLN female (*n =* 8 at 18 months and *n =* 7 at 24 months) and NLL female (*n =* 6 at 18 months and *n =* 1 at 24 months), using two-way ANOVA followed by Tukey’s post hoc test (**a**,**d**). * *p* ≤ 0.05 in NNN (24-month-old data were excluded from the analysis because there was only one mouse in the NLL group). An unpaired *t*-test was used to compare NNN and NLN, while NLL was excluded from the analysis because there was only one sample (**b**,**c**,**e**,**f**). *** *p* ≤ 0.001.

**Figure 5:**
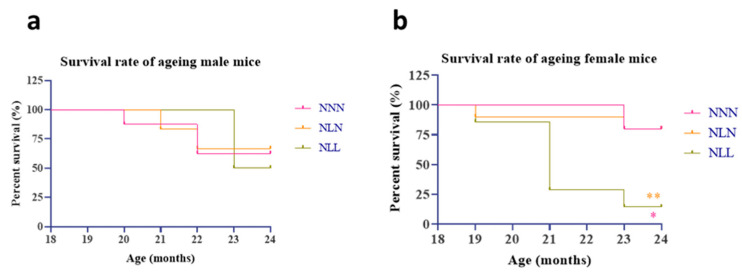
The effect of an LPD during lactation on the survival rate of male (**a**) and female (**b**) progenies. NNN (*n =* 8), NLN (*n =* 6), NLL (*n =* 4) at 18 months of age for males and NNN (*n =* 5), NLN (*n =* 10), NLL (*n =* 7) at 18 months of age for females. Log-rank test, three-group analysis. * *p* ≤ 0.05 for NLL compared to NNN, and ** *p* ≤ 0.01 for NLL compared to NLN.

The data were analysed using GraphPad Prism 8 (version 8.02) and presented as mean ± standard error of the mean (SEM). The Student’s *t*-test was used to compare NN and NL groups at 21 days of age (Figure 2, Figure 3 and Figure 4), and one-way ANOVA was used to compare NNN, NLN and NLL at 3, 18 and 24 months of age, followed by Tukey’s post hoc analysis comparing all groups (Figure 2, Figure 3 and Figure 4). One-way ANOVA was used to compare the difference between every age group of NNN, NLN and NLL separately, followed by Tukey’s post hoc analysis comparing all groups (Figure 2, Figure 3 and Figure 4). The chi-square test was used to compare the distributions of myofibres (Figure 3e–j) and 2-way ANOVA to compare the myofibres’ CSA range, followed by Tukey’s post hoc analysis (Figure 3e–j and Figure 4a–d). Only one NLL female mouse survived up to 24 months of age; therefore, no statistical analysis was performed for this group. An unpaired *t*-test was used to compare the grip strength of NLN to NNN, and NLL was excluded in this analysis due to the small sample size (Figure 4b,c,e,f). Finally, the Log-rank test, a three-group analysis, was used to compare the lifespan of NLN and NLL to NNN (Figure 5). The data were considered statistically significant with a *p*-value less than 0.05.

## 3. Results

### 3.1. LPD’s Effect on Lifespan

A postnatal LPD significantly decreased the lifespan of female mice (Figure 5b). Protein restriction during lactation followed by a normal diet after weaning (NLN) did not have a major effect on the survival rate of either male or female (Figure 5a,b) offspring. There was no difference in the survival rate between NNN, NLN and NLL groups in male mice (Figure 5a).

### 3.2. Lifelong Effects of Lactational LPD on Body Weight and TA Muscle Weight of Male and Female Mice

A lactational low-protein diet significantly affected the body weight and muscle weight of male and female mice. A protein-deficient diet during lactation led to a decrease in the weight of both male and female mice at weaning age (NL-21 days) (Figure 2a,d). The male mice fed an LPD post-weaning (NLL) maintained a significantly lower body weight at 3 months and 18 months of age compared to NNN and NLN mice. At 24 months, NLL male mice had a non-significant lower body weight since NNN and NLN mice also significantly lost weight. The male NLN mice caught up with NNN mice at 3 months of age, but they showed accelerated weight loss during ageing in comparison to NNN mice (Figure 2a). On the other hand, NLN female mice also caught up with NNN female mice at 3 months of age, while NLL female mice had a similar weight as NNN mice at 18 months of age (Figure 2d).

*TA* weight in male mice showed a similar pattern as the body weight (Figure 2b). NL male mice had a lower *TA* weight at 21 days of age; the mice maintained a low *TA* weight. At 24 months, the weight difference between NLL and NNN was not significant because *TA* weight of NNN male mice significantly decreased. The *TA* weight of NLN male mice caught up with that of NNN mice at 3 months of age; however, both showed an accelerated loss of weight during ageing. NL male mice had a lower ratio of muscle weight to body weight (index) at 21 days of age (Figure 2c). However, this difference was no longer present at 3 months, indicating that the loss of muscle weight was in proportion to body weight. During ageing, at 18 and 24 months of age, the index of all male mice decreased in comparison to 3 months of age. Although not significant, NLN male mice had a lower index than NNN and NLL mice, potentially indicating a higher loss of muscle mass.

The diet affected female mice differently from male mice in terms of changes in muscle. At 21 days of age, the *TA* weight was lower in the NL group (Figure 2e). However, in both NLN and NLL mice, this difference was diminished as compared to NNN mice at 3 months of age and then showed an accelerated muscle weight loss during ageing (Figure 2e). These data suggest that short-term and long-term LPDs negatively affect the muscle weight of female mice during ageing. Moreover, NL female mice have a lower muscle-to-body weight index at 21 days. However, both males and females show similar rates of muscle wasting at 3 months of age when adjusted for body weight (Figure 2f).

### 3.3. Lifelong Effect of LPD during Lactation on Myofibres of TA Muscle in Male and Female Mice

**Figure 6:**
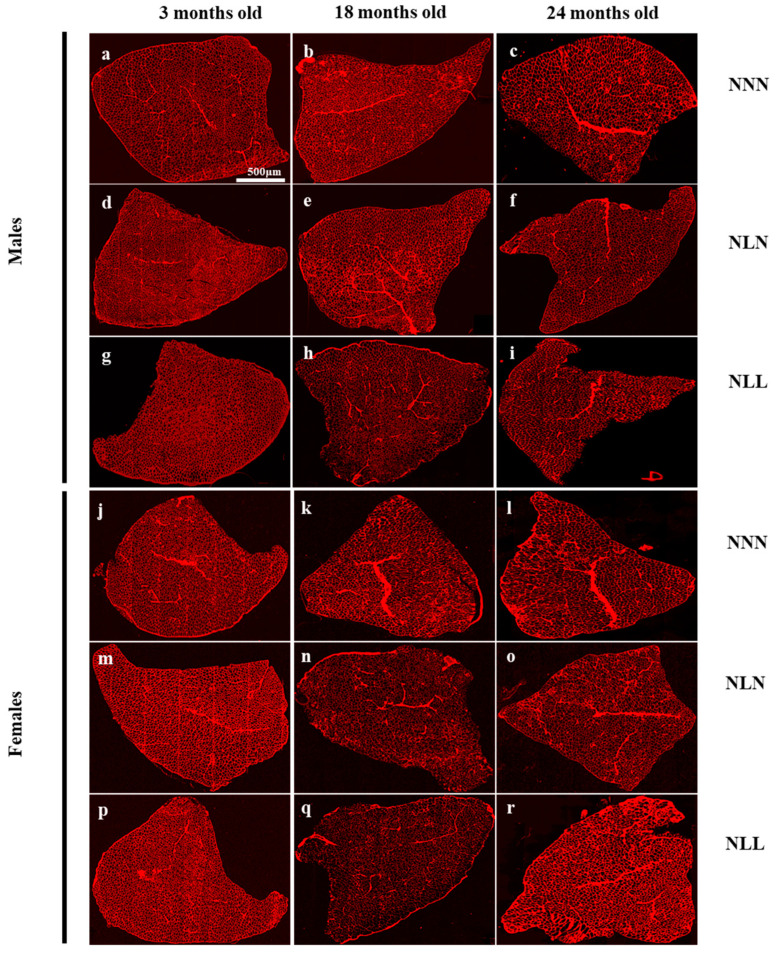
Representative cross-sections of TA muscle stained with WGA and imaged with Z1 digital slide scanner of males (**a**–**i**) and females (**j**–**r**). Cross-section of TA of NNN male at 3 months (**a**), 18 months (**b**) and 24 months (**c**) of age. Cross-section of TA of NLN male at 3 months (**d**), 18 months (**e**) and 24 months (**f**) of age. Cross-section of TA of NLL male at 3 months (**g**), 18 months (**h**) and 24 months (**i**) of age. Cross-section of TA of NNN female at 3 months (**j**), 18 months (**k**) and 24 months (**l**) of age. Cross-section of TA of NLN female at 3 months (**m**), 18 months (**n**) and 24 months (**o**) of age. Cross-section of TA of NLL female at 3 months (**p**), 18 months (**q**) and 24 months (**r**) of age. Magnification: 20×. Scale: 500 µm.

Quantitative measurements of CSA and fibre numbers were performed using histological sections of TA muscles. Representative sections for each group are presented in Figure 6.

Histological analysis of *TA* muscle showed that the number of myofibres in the male offspring of NNN and NLN mice significantly decreased at 18 months of age in comparison to 3 months (Figure 3a), while NLL mice showed a steady decrease in the number of fibres at 18 and 24 months of age (Figure 3a). The comparison between NNN, NLN and NLL mice at 3, 18 and 24 months of age showed that there was no significant difference between them at any of the timepoints (Figure 3a).

The CSA of the myofibres was significantly lower in NLL male mice in comparison to NLN and NNN mice at 3 months of age (Figure 3b). During ageing, the CSA was not different among the groups, which can be due to the increase in the CSA of the myofibres of NLL mice at 18 months of age. NNN and NLN were similar in terms of myofibre number and CSA. The distributions of myofibres of NNN and NLN were very similar, while the NLL group had more fibres of a smaller range at 3 months of age. During ageing, the distribution of myofibres of NLL was similar to that of NNN and NLN (Figure 3e–g), which correlates with the increase in the CSA of NLL myofibres at 18 months of age in comparison to 3 months of age (Figure 3b).

Similarly to those of the males, fibre number, CSA and fibre distribution in NLN females were similar to those of NNN (Figure 3c,d,h–j). However, NLL female mice had a lower number of fibres at 3 months of age compared to NNN and NLN, and the number was preserved during ageing (Figure 3c). At 18 months of age, the number of myofibres of NNN and NLN mice decreased and had a lower number than NLL female mice. An LPD did not have a significant effect on the CSA of the myofibres or the distribution of the myofibres in females (Figure 3d,h–j). However, even though not significant, both NLN and NLL had a lower CSA than NNN at 24 months of age. Only one NLL mouse survived until 24 months of age (Figure 5b); therefore, no statistical analysis was possible.

### 3.4. Effect of LPD during Lactation on the Grip Strength of Ageing Male and Female Mice

A lactational LPD negatively affected the grip strength of male offspring. The maximum hanging time measured in this study fluctuated throughout the measurements, which could have been due to the weight or behavioural factors while conducting the experiment. The hanging times of different groups of males and females were not significantly different between groups (Figure 4a,b,d,e). Holding impulse was calculated, taking into consideration the body weight of the mice. The holding impulse of NLN male mice at 24 months normalised to 18 months was significantly lower than that of NNN mice (Figure 4c). The holding impulse of NLL mice was also lower than that of NNN mice; however, there was only one mouse in the group because the mice were terminated before the end of the experiment; therefore, statistical analysis was not possible for the NLL group. This suggests a higher decline rate in grip strength in NLN and NLL mice compared to NNN mice. The decline in NLN male mice might be due to their body weight, as the change was only seen in holding impulse and not hanging time. The holding impulse of female mice was not different among the groups, suggesting that body weight did not affect the grip strength of female mice (Figure 4f).

## 4. Discussion

The aim of this study was to examine the impact of a maternal low-protein diet during lactation on the development and ageing processes of skeletal muscle in male and female offspring. Various parameters, including body weight, muscle weight, grip strength and survival rate, were evaluated at different life stages: weaning (21 days), early adulthood (3 months), early ageing (18 months) and late ageing (24 months). Furthermore, histological analyses of the *TA* muscle in the progenies were conducted. Additionally, we examined whether this dietary intervention, followed by either a sustained LPD or a transition to an NPD, contributed to the early onset of sarcopenia. All mice were fed ad libitum; therefore, the quantity of food was not controlled during this experiment.

An LPD during lactation did not increase the lifespan of male and female progenies. In this study, the survival rate of aged female NLL mice was significantly lower compared to that of NLN and NNN mice. Although the survival rate of male NLL mice decreased by 50%, this decrease was not statistically significant when compared to that of NNN and NLN mice. On the other hand, the survival rate of NLN male and female mice was not affected by diet and remained similar to that of NNN mice. Previous studies have suggested that a maternal LPD and slow growth rate may extend the lifespan of rodent progenies [27,28,36,37]. Both Heppolette et al. (2016) and Ozanne and Hales (2004) used the same diet as the one used in this study and focused on the effect of a lactational low-protein diet on male offspring. In all these studies, the offspring remained smaller post-weaning, in contrast to the current study, where recuperation of weight was observed. Ozanne and Hales (2004) focused on the effect of slow growth rate postnatally due to an LPD during lactation corrected post-weaning and found that it increases the lifespan of male pups. Heppolette et al. (2016) correlated this increase in lifespan with delayed ageing of the adaptive immune system. Guzmán et al. (2006) studied the effect of a maternal low-protein diet (10%) on female progenies in rats and found that the survival rate of NLN mice was not significantly lower than NNN mice even though they had only one mouse at 22 months of age. None of these studies had an NLL group in their studies. However, Carey et al. (2022) negatively correlated protein intake with the lifespan of male and female *Drosophila melanogaster,* which contradicts this study [38].

The impact of maternal LPD during lactation on body weight, muscle weight, grip strength and survival rate differed between male and female offspring. In this study, 21-day-old male and female mice that were fed an LPD during lactation (NL) exhibited a lower body weight compared to control mice (NN). This finding aligns with previous studies that have demonstrated a negative correlation between an LPD during lactation and the body weight of offspring [16,17,25,27]. Interestingly, the body weight of male NL mice that were subsequently fed an NPD post-weaning (NLN) caught up with the body weight of NNN mice at 3 months of age and exhibited a growth pattern similar to the control group throughout the ageing process. Conversely, NLL male mice consistently maintained a lower body weight throughout their lifespan compared to both NNN and NLN mice. In the case of female mice, a post-weaning LPD resulted in a different pattern compared to males. NLN and NLL female mice caught up with NNN mice in terms of body weight at 3 months and 18 months of age, respectively. This finding contrasts with the results of Heppolette et al. (2016), which showed that NLN male mice consistently maintained a lower body weight than NNN mice throughout their lifespan and did not show any data about females. Various studies have consistently demonstrated the negative effects of nutritional restriction on the body weight and growth rate of male progenies [12,39]. Catch-up growth following in utero growth restriction as a result of improvements in nutritional deficits during early life stages has been reported [27,36]. An LPD during adulthood has also been linked to a decrease in body weight due to metabolic alterations [40], which provides an explanation for the lower body weight observed in NLL mice.

A postnatal LPD affected the *TA* muscles of female and male progenies differently. Both males and females showed a lower *TA* weight at 21 days of age. However, in female offspring, by 3 months of age, their *TA* weight caught up to that of the control group regardless of the post-weaning diet. At 18 months of age, there was a decrease in *TA* weight, accompanied by a significant loss of muscle fibres in one group (NLN), resembling the pattern observed in control mice. The other group (NLL) did not experience fibre loss but showed a potential higher number of smaller range fibres. Additional factors such as fat infiltration, connective tissue changes, water retention or fibre remodelling may contribute to the observed *TA* weight changes in NLL mice [41]. In male offspring, the *TA* weight of NLN mice caught up to that of the control group by 3 months of age, and a significant decrease was observed at 24 months of age. NLL male mice consistently had lower *TA* weight throughout their lifespan. Histological analysis indicated that an LPD did not significantly affect the number of myofibres but had a negative impact on myofibre CSA in NLL mice at 3 months of age. Interestingly, the myofibre CSA increased during the ageing process, potentially due to the loss of smaller range fibres. The exact mechanism behind the *TA* weight loss in NLL male mice requires further investigation, potentially related to fat infiltration or connective tissues. Furthermore, it is worth mentioning that the analysis at 24 months of age for males and females essentially compares the fittest progenies that survived up to that age.

An LPD is known to inhibit protein synthesis and can lead to protein catabolism in skeletal muscle when associated with a high-carbohydrate diet [42]. In humans, an LPD was positively associated with muscle mass loss [43,44]. Previous studies showed that a lactational LPD in male progenies led to a decrease in skeletal muscle weight at 21 days of age and 3 months of age for NLN mice in comparison to a control [25,27]. In rats, an LPD affected the triacylglycerol and glycogen levels in skeletal muscle but had no effect on skeletal muscle mass and fibres [16]. These studies showed different effects of an LPD on skeletal muscle, but none of them studied the effect of a lactational LPD during ageing or a postnatal LPD. During ageing, skeletal muscle loss occurs [45], which is consistent with NNN and NLN mice in this study but not with NLL. This suggests that protein restriction during lactation, normalised during adult life, does not affect muscle mass. The *TA* weight of both male and female NLL mice was maintained during ageing; the mechanism behind this stability in *TA* weight needs to be further investigated. The long-term protein restriction in both male and female progenies prevented the muscle mass loss associated with ageing. Skeletal muscles are mainly made of myofibres that can affect muscle mass. However, skeletal muscle also contains fat and connective tissue that can also affect its mass. An LPD was associated with protein degradation and loss of myofibres due to metabolic changes caused by a low-protein diet during adulthood [40]. Surprisingly, in this study, the number of myofibres of the *TA* muscle of mice fed an LPD postnatally (NLL) was maintained during ageing. An LPD was also associated with a switch from type II to type I myofibres, which are smaller than type II [24]. This switch in myofibre types is also associated with ageing [46,47]. Unfortunately, in this study, the fibre type of the *TA* muscle was not assessed; however, the data showed that an LPD affects CSA in NLL male mice at 3 months, which can be associated with myofibre remodelling. In females, even though not significant, NLL mice had a lower CSA; however, only one mouse survived until 24 months of age. Male and female mice are known to have different skeletal muscle composition, contractility and metabolism [48]. Sex hormones also affect skeletal muscle physiology. For example, oestrogen, which is a female hormone, is associated with skeletal muscle maintenance and high muscle regeneration, while testosterone, a male hormone, is associated with muscle growth [49]. Zambrano et al. (2005) showed that NLN rats had an increased testosterone level at early stages of life, but after 6 months, testosterone level decreased, which might explain the decrease in *TA* muscle weight in NLN during ageing and the catch-up growth of *TA* muscle at 3 months of age [50]. A maternal LPD during lactation was associated with a decrease in oestrogen level, which delays the development of reproductive organs and induces early ageing of the reproductive system [37,51]. The low oestrogen level, which was associated with a lactational LPD, was also associated with muscle loss, especially during menopause in females, which contradicts the data in this study. Further investigation is needed to understand the underlying mechanism of muscle loss and maintenance in male and female mice.

A lactational LPD led to a decrease in the holding impulse of old male mice but did not have an effect on the grip strength of female mice. The decline in muscle strength along with muscle mass is characteristic of sarcopenia [52]. The decrease in muscle activity was associated with changes in muscle physiology. For example, myofibre size and myofibre type affect physical activity and muscle strength. Protein restriction was also associated with a decrease in physical performance [12,22,24,53].

### Limitations of the Study

During lactation, the number of pups was maintained similar between all groups. Following weaning, mice were housed and maintained with their litter mates until culling. Mice were fed ad libitum, but because mice were housed in groups, it was not possible to monitor or control food consumption per mouse. In some cases, mice developed illnesses during ageing and had to be humanely killed. Hence, the number of mice, particularly during ageing, was different in some groups (for example, in the NLL group); this could also have affected the food consumption and activity of mice in different cages.

Only the TA muscle (primarily type II) was analysed in this study. This means that type I muscles, such as the soleus muscle, could have presented a different outcome. However, the comparison of type I versus type II muscles was not within the scope of this study.

The grip strength was measured using the hanging method only, instead of other methods such as IMADA, and was only performed in old mice (>18 months old). We preferred to use this method to avoid causing further stress to mice during old age. Finally, as this was an observational study, no biochemical analyses were performed.

## 5. Conclusions

In conclusion, the findings of this study highlight the impact of a lactational LPD on the development and ageing of male and female offspring. Firstly, it was observed that mice fed an LPD during lactation had a lower body weight compared to control mice. However, NLN male mice eventually caught up with their counterparts fed a normal-protein diet, indicating a compensatory growth response. Unlike males, female NLN and NLL mice eventually caught up with NNN mice. Furthermore, the effects of the LPD on the *TA* muscle differed between male and female mice. In male mice, the LPD primarily affected the size of myofibres of the *TA* muscle at 3 months of age but not during ageing. On the other hand, in female mice, the LPD predominantly influenced the number of myofibres in the *TA* muscle. Notably, a significant finding was the impact of a postnatal LPD on the survival rate of female progenies. These results shed light on the importance of proper nutrition during lactation and its potential implications on the ageing of offspring. Additional research is needed to understand the underlying mechanisms involved in the loss or preservation of muscle mass during ageing, as well as the distinct mechanisms operating in male and female offspring.

## Data Availability

Data is contained within the article.
